# Large Language Model–Based Identification of Subjective Cognitive Decline in Electronic Health Records Among Older Adults With Normal Cognitive Testing: Retrospective Cohort Study

**DOI:** 10.2196/93011

**Published:** 2026-10-07

**Authors:** Liqin Wang, Rebecca E Amariglio, Sheril Varghese, Jiazi Tian, Diane Seger, Li Zhou, Gad A Marshall

**Affiliations:** 1 Department of Medicine, Mass General Brigham, 399 Revolution Drive, Somerville, MA, 02145, United States, 857-282-4089; 2 Harvard Medical School, 25 Shattuck Street, Boston, MA, 02115, United States; 3 Department of Neurology, Mass General Brigham, Boston, MA, United States; 4 Columbia University Irving Medical Center, New York, NY, United States; 5 Department of Medicine, Massachusetts General Hospital, Boston, MA, United States; 6 Research Information Science & Computing, Mass General Brigham, Boston, MA, United States

**Keywords:** large language model, cognitive concern, subjective cognitive decline, electronic health records, documentation

## Abstract

**Background:**

Subjective cognitive decline (SCD) typically refers to self- or informant-reported decline in cognition despite the absence of objective impairment on standardized testing. Older adults with documented normal cognitive test performance provide a pragmatic anchor cohort for electronic health record (EHR)–based SCD phenotyping. However, cognitive concerns are primarily recorded in unstructured notes and are inconsistently documented, making it unclear how often and in whom concerns are captured in routine care.

**Objective:**

This study aims to operationalize EHR-based SCD phenotyping in an objectively normal-testing cohort using a large language model (LLM)–based natural language processing approach and to examine the frequency and clinical and sociodemographic correlates of cognitive concern documentation.

**Methods:**

We conducted an EHR-based observational study of patients aged 65 years or older with a first normal cognitive test recorded in EHR flowsheets between January 2019 and April 2024 in a large health care system. We developed and iteratively refined a 2-stage LLM-based natural language processing pipeline to identify documented cognitive concerns in unstructured notes during the 12 months prior to the index date, and evaluated performance against manual review. We quantified the frequency of documented concerns within this normal-testing cohort and used multivariable logistic regression to assess associations with sociodemographic factors (age, marital status, insurance, and neighborhood Area Deprivation Index), sequentially adjusting for clinical comorbidities and relevant medications.

**Results:**

Among 15,750 older adults with normal cognitive test scores, 13.8% (n=2175) had at least 1 documented cognitive concern in the prior year, captured in 1.2% (7394/605,177) of notes. On manual validation, the 2-stage pipeline (Med42-v2-8B screening followed by GPT-4o confirmation) achieved a sensitivity of 0.957, positive predictive value of 0.935, specificity of 0.985, and *F*_1_-score of 0.945 for cognitive concern identification. Documentation was more likely in older individuals, those with commercial (vs Medicare) insurance, and those with neurological and psychiatric conditions. In fully adjusted models, Parkinson disease (adjusted odds ratio [aOR] 5.29, 95% CI 3.60‐7.77), traumatic brain injury (aOR 4.63, 95% CI 3.15‐6.81), stroke or transient ischemic attack (aOR 3.46, 95% CI 3.15‐4.18), epilepsy (aOR 2.52, 95% CI 1.83‐3.49), depression (aOR 1.54, 95% CI 1.36‐1.75), and excessive alcohol use (aOR 1.48, 95% CI 1.06‐2.06) were among the strongest correlates of cognitive concern documentation. In contrast, obesity (aOR 0.73, 95% CI 0.65‐0.82), hyperlipidemia (aOR 0.59, 95% CI 0.51‐0.67), and residence in more deprived neighborhoods (higher Area Deprivation Index) were associated with lower odds of documented cognitive concerns.

**Conclusions:**

A 2-stage LLM pipeline enabled accurate identification of documented cognitive concerns consistent with SCD among older adults with normal cognitive testing. Documentation was uncommon and selectively captured by clinical and sociodemographic factors, with implications for equity and the validity of EHR-based phenotypes.

## Introduction

Given emerging therapies for Alzheimer disease (AD), early detection is increasingly critical to ensure interventions are delivered at the most beneficial stage of the disease trajectory. Subjective cognitive decline (SCD) is characterized by self- or partner-reported concerns about memory or other cognitive functions despite normal performance on standardized cognitive tests [1]. SCD represents one of the earliest stages at which cognitive changes become noticeable to patients, family members, or clinicians, often preceding measurable impairment, such as mild cognitive impairment (MCI) [2,3]. Importantly, longitudinal meta-analyses show that SCD is associated with approximately 2-fold higher risk of subsequent dementia and elevated risk of incident MCI, supporting SCD as a clinically meaningful risk marker rather than a universally benign complaint [4,5]. As such, recognizing SCD in clinical settings provides a valuable window of opportunity for early intervention, risk reduction, and enrollment in clinical trials targeting preclinical AD [6].

However, SCD remains underrecognized in routine care and is difficult to identify systematically in electronic health records (EHRs). Unlike MCI or dementia, SCD lacks a standardized diagnostic code, limiting scalable identification for clinical screening and research. In practice, documented cognitive concerns and objective testing are often misaligned: many individuals never undergo formal cognitive testing, while others are tested without any documented concerns [7,8]. Among individuals with normal test scores, the presence vs absence of documented cognitive concerns provides a clinically relevant contrast for examining how early symptoms are recognized and recorded. Characterizing variation in documentation by clinical and sociodemographic characteristics is critical for equitable early detection and for valid EHR-based cognitive aging research. Emerging evidence suggests that recognition, reporting, and clinical documentation of cognitive concerns may vary across socioeconomic, racial or ethnic, and health care access contexts, potentially introducing bias into EHR-based phenotyping and delaying identification of individuals at risk for future cognitive decline [9]. Individuals from disadvantaged communities may also face barriers to early evaluation and specialty memory care, further affecting how cognitive concerns are documented in routine clinical practice [10,11].

Cognitive concerns are typically embedded in free-text clinical notes rather than structured fields, making automated detection challenging, particularly given the wide variation in language describing cognitive concerns among clinicians, patients, and family members [12,13]. Recent advances in large language models (LLMs) present an opportunity to overcome these barriers. LLMs have significantly enhanced the depth, accuracy, and efficiency of natural language processing (NLP) for automated information extraction from EHR data. Unlike traditional rule-based NLP tools that rely on predefined patterns and keywords, LLMs are trained on vast text corpora to learn statistical relationships in language, enabling them to capture context, nuance, and phrasing variation beyond the capabilities of rule-based systems. LLMs can analyze unstructured notes at scale, searching for nuanced mentions of cognitive concerns that might otherwise go unnoticed. Although our prior studies have used deep learning and LLMs to detect broad indicators of cognitive decline in EHRs [14], few studies have focused on SCD, and fewer have compared individuals with and without SCD among those with normal cognitive test performance [12,13]. Moreover, factors that may affect the documentation of subjective concerns, such as comorbidities, medication use, and sociodemographic characteristics, remain incompletely understood.

In the present study, we leveraged LLM and EHR data to systematically identify SCD among individuals with normal cognitive test performance. The aims of this study were to (1) develop and evaluate a scalable LLM-based approach for identifying documented SCD from unstructured clinical notes and (2) compare sociodemographic, clinical, and medication characteristics of individuals with and without documented SCD. We hypothesized that individuals with documented SCD would differ in clinical and sociodemographic profile despite normal cognitive testing.

## Methods

### Data Sources

We used EHR data from Mass General Brigham (MGB), a large integrated health care system that includes Massachusetts General Hospital, Brigham Women’s Hospital, and affiliated community hospitals in Massachusetts. Data were obtained from 2 enterprise EHR data repositories, including the Research Patient Data Registry (RPDR) and the Enterprise Data Warehouse (EDW), spanning from January 1, 2015, to April 10, 2024.

### Ethical Considerations

The study was approved by the MGB Institutional Review Board. Cognitive assessment data were collected as part of routine clinical care and obtained retrospectively from the EHR. The study team did not administer any cognitive assessment instruments for research purposes, and licensing information was not available through the EHR repositories.

### Study Sample

We defined presumed SCD as the presence of documented cognitive concerns among individuals with normal cognitive test scores. Individuals were initially identified based on cognitive assessments recorded in EHR flowsheets between January 1, 2015, and April 10, 2024. Cognitive assessments included the Mini-Cog, Mini-Mental State Examination (MMSE), Montreal Cognitive Assessment (MoCA), Saint Louis University Mental Status (SLUMS) examination, and the Ascertain Dementia 8-Item Informant Questionnaire (AD8) dementia screening interview. When all MMSE items were documented, but the total score was missing, we calculated the total by summing item scores. Cognitive assessments without a total score were excluded, including partly administered tests due to time constraints.

To establish a baseline of normal cognition, we defined the index data as each individual’s first cognitive assessment occurring between January 1, 2019, and April 10, 2024. The period from January 1, 2015, to December 31, 2018, served as a lookback window to identify prior cognitive assessments. Individuals with any cognitive assessment recorded during this earlier period were excluded to ensure that the index assessment represented the first observed cognitive assessment in the EHR during the study period. We further restricted the cohort to individuals aged ≥65 years at the time of their first assessment to focus on age groups most relevant to AD and related dementias.

We narrowed down the study cohort to include individuals with normal cognitive test scores based on established cutoff criteria: Mini-Cog: 4‐5, MMSE: ≥26, MoCA: ≥26, SLUMS: ≥27, AD8: 0‐1. For the remaining individuals, we extracted all their clinical notes within 1 year prior to the initial cognitive test from MGB’s EHR databases. Individuals who did not have notes available in the MGB EHR were excluded. We further excluded individuals who had a prior history of cognitive decline, related diagnosis (eg, MCI or dementia) prior to the index date. Specifically, we excluded individuals with evidence of prior cognitive decline or related diagnoses before the index date. Prior history was identified using structured EHR data extracted from RPDR and EDW, including diagnosis records and problem list entries, based on relevant *International Classification of Diseases* (*ICD*) codes for dementia, MCI, AD, and related cognitive disorders documented before the index cognitive assessment date. In addition, note-derived cognitive concern information (see *LLM-Based Cognitive Concern Extraction* section) was used to identify prior patient-related cognitive decline documentation, including cognitive assessments, MCI, and dementia documentation in the notes. Individuals meeting any of these criteria before the index date were excluded from the final cohort.

### Dependent Variable: Cognitive Concerns

The primary dependent variable was the presence of cognitive concerns within one year prior to the first normal cognitive assessment, defined as a binary indicator (yes/no). Given that cognitive concerns in the early stages of cognitive decline were often documented in free-text, we used LLMs to identify and extract cognitive concerns from clinical notes (described in the *LLM-Based Cognitive Concern Extraction* section).

### LLM-Based Cognitive Concern Extraction

We used a combination of open-source and commercial LLMs to extract cognitive concerns from clinical notes. Notes were first split into smaller sections of up to 300 words. For each section, the model was prompted to (1) indicate whether a cognitive concern is mentioned (yes/no) and (2) extract the relevant text verbatim (see Table S1 in Multimedia Appendix 1 for prompts). As the open-source model, we selected Med42-v2-8B, a Llama 3-based model fine-tuned on clinical data, which enables efficient processing of large volumes of notes at minimal cost [15]. The prompt of this model was optimized for high sensitivity to exclude cases that clearly lacked cognitive concerns while capturing as many potential positives as possible. As the commercial model, we deployed OpenAI GPT-4o in a Health Insurance Portability and Accountability Act (HIPAA)-compliant Microsoft Azure environment. We adopted a 2-stage pipeline to balance scalability and accuracy. In iterative prompt development, Med42-v2-8B was effective as a high-sensitivity screening model but produced more false positives, including occasional overinterpretation or hallucination of cognitive concerns. More restrictive prompts improved precision but reduced sensitivity and did not fully eliminate these errors. Therefore, Med42-v2-8B was used to screen out notes unlikely to contain cognitive concerns, and GPT-4o was applied only to Med42-v2-8B-positive notes to improve confirmation accuracy while reducing computational cost.

### Evaluation of LLMs in Cognitive Concern Extraction

To evaluate cognitive concern identification, we constructed an evaluation set of 500 note chunks using stratified sampling by model-predicted class. Because the prevalence of cognitive concerns in the full corpus was low, we randomly sampled note chunks classified as positive and negative by the Med42-v2-8B model at a 3:1 ratio to enrich positive cases. Two annotators (JT and SV) independently reviewed an initial subset of 100 note chunks to assess interannotator agreement for binary cognitive concern identification using Cohen κ. After substantial agreement was achieved (κ=0.70; overall agreement=90%), the annotators independently reviewed the remaining note chunks, with each annotator reviewing an additional 200 chunks. Discrepancies between annotators were adjudicated by a cognitive neurologist (GAM) to establish the reference standard. In addition, selected discordant cases between LLM predictions and reference labels were reviewed by domain experts to better characterize sources of disagreement and model errors. We then compared the LLM outputs with this reference standard and assessed performance using sensitivity, specificity, positive predictive value (PPV), and *F*_1_ score (the harmonized score of sensitivity and PPV).

### Covariates

Individual-level covariates included age (in years), patient-reported sex, race or ethnicity, marital status, education level, language preference, and insurance type. Neighborhood deprivation was assessed using the 2023 Area Deprivation Index (ADI) [16]. For each individual, we derived a 9-digit ZIP code from the residential address and ZIP code, then linked this to the ADI database to obtain the national ADI percentile. Individuals were categorized into three groups based on their national ADI percentile: low (1st-20th percentile), medium (21st-40th percentile), and high (41st-100th percentile).

To account for documentation opportunity, we calculated the number of clinical notes in the year prior to the index date. To capture comorbidity burden, we identified relevant medical conditions across domains like neurological, cardiovascular, cerebrovascular, metabolic, endocrine, psychiatric, autoimmune, infectious diseases, and sensory impairment (eg, hearing and visual loss), as well as lifestyle factors (eg, smoking) [17]. Conditions were identified using rule-based phenotyping algorithms tailored to individual conditions (see Table S2 in Multimedia Appendix 1). We also calculated the Charlson Comorbidity Index using *ICD* diagnosis codes recorded in the 2 years prior to the index date.

In addition, we reviewed the medication history within 1 year prior to the cognitive tests based on prescription records. Medications were grouped into categories based on their potential cognitive effects, including anticholinergic medications, benzodiazepines and sedative-hypnotics, antidepressants, antipsychotics, antiepileptics, opioids, and other medications of concern (eg, beta-blockers, corticosteroids, chemotherapy agents, and H2-receptor antagonists; Table S3 in Multimedia Appendix 1). Each class includes medications known to impair cognition through mechanisms such as anticholinergic burden, GABAergic activity, sedative properties, or blood-brain barrier penetration.

### Statistical Analysis

We compared demographic and clinical characteristics between individuals with and without documented cognitive concerns. For continuous variables, we used 2-tailed *t* tests; for categorical variables, we used the chi-square tests or Fisher exact tests. To identify factors associated with documented cognitive concerns, we fitted multivariable logistic regression models with cognitive concern (yes/no) as the dependent variable. For the number of clinical notes in the year prior to the index date, we modeled it as a log2-transformed variable to address right-skew and facilitate interpretation (ie, per doubling of note count). Three nested models were constructed: Model 1 included sociodemographic variables and log2-transformed note count; model 2 additionally included clinical conditions (eg, Charlson Comorbidity Index and selected comorbidities); and model 3 further included medication classes based on medication prescriptions (eg, anticholinergics and antidepressants). Variables with *P*<.05 in univariate analyses were considered for inclusion in multivariable models. We reported crude and adjusted odds ratios (aORs) with 95% CIs. All analyses were conducted in R (version 4.4.1; R Foundation for Statistical Computing) using RStudio.

## Results

We identified 57,474 individuals with cognitive assessments documented in EHR flowsheets (Figure 1), of whom 52,862 had total scores available. We then restricted the cohort to 48,146 individuals whose first cognitive test occurred between January 1, 2019, and April 10, 2024, and further to 42,370 individuals aged 65 years or older. Limiting the cohort to those with normal cognitive scores yielded 16,477 individuals. After excluding individuals with prior cognitive decline or earlier cognitive assessments, 15,750 individuals met all inclusion and exclusion criteria.

Among the final study cohort, 13.8% (n=2175) had a documented cognitive concern in the prior year. The cognitive concerns were identified from 7395 of 605,177 (1.2%) notes recorded in the year before the first normal cognitive test. The Med42-v2-8B model showed high sensitivity (97.8%) but moderate PPV (64.7%) for identifying cognitive concerns (Table S4 in Multimedia Appendix 1). When GPT-4o was used to reprocess all positive cases identified by the Med42-v2-8B, sensitivity remained high (95.7%) while PPV increased to 93.5% and specificity to 98.5%, improving the *F*_1_-score from 0.779 to 0.945 and yielding more balanced performance across all metrics. We manually reviewed false positives and false negatives from the final pipeline. Most false positives reflected ambiguous or inferential mentions, often related to delirium or other acute conditions, whereas false negatives typically involved very subtle or indirect references to cognitive symptoms (eg, mentions of a “memory unit”) and institutional abbreviations or shorthand.

Most index cognitive assessments occurred in outpatient primary care settings (14,427/15,750, 91.6%), followed by hospital or rehabilitation settings (460/15,750, 2.9%) and outpatient neurology or geriatrics settings (440/15,750, 2.8%; Table S5 in Multimedia Appendix 1). Cognitive concern documentation was distributed across multiple clinical settings, including hospital or rehabilitation settings (574/2175, 26.4% patients), outpatient primary care (1045/2175, 48.0%), neurology or geriatrics (257/2175, 11.8%), and medical specialties (215/2175, 9.9%; Table S6 in Multimedia Appendix 1). These findings suggest that cognitive concerns were captured across diverse clinical contexts rather than solely in primary care or neurology encounters. Detailed score distributions by cognitive test are provided in Table S7 in Multimedia Appendix 1.

Among these 15,750 individuals, 15,038 (95.5%) were White, only 228 (1.4%) identified as Hispanic, and 9651 (61.3%) were female (Table 1). Most were married (n=10,095, 64%). More than half of the cohort (n=8115, 51.5%) had a college education or higher, and 97.9% (n=15,426) reported English as their preferred language. The majority (n=12,202, 77.5%) had Medicare as their insurance, while 3031 (19.2%) had commercial insurance. In terms of neighborhood socioeconomic status, 8278 (52.6%) lived in the least deprived area with national ADI≤20.

**Figure 1.**
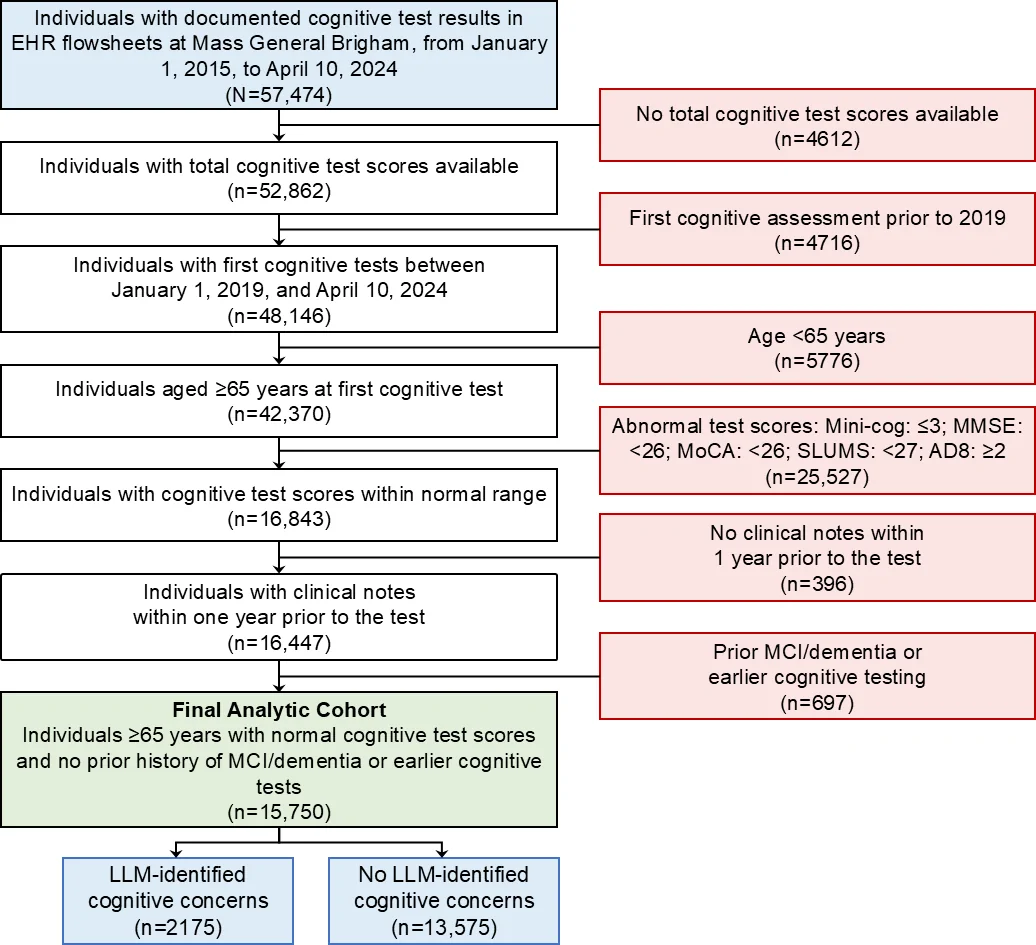
Cohort derivation and classification of patients with and without large language model (LLM)-identified cognitive concerns. AD8: Ascertain Dementia 8-Item Informant Questionnaire; EHR: electronic health record; MCI: mild cognitive impairment; MMSE: Mini-Mental State Examination; MoCA: Montreal Cognitive Assessment; SLUMS: Saint Louis University Mental Status Examination.

**Table 1.** Demographics and cognitive characteristics of individuals with normal cognitive test scores, stratified by presence of cognitive concerns.

Characteristics	Overall (N=15,750)	With cognitive concerns (n=2175)	Without cognitive concerns (n=13,575)	*P* value^a^
Age (y), mean (SD)	73.5 (6.4)	75 (6.9)	73.2 (6.3)	<.001
Sex (female), n (%)	9651 (61.3)	1298 (59.7)	8353 (61.5)	.10
Race, n (**%**)				.36
White	15,038 (95.5)	2068 (95.1)	12,970 (95.5)	
Non-White	712 (4.5)	107 (4.9)	605 (4.5)	
Black	191 (1.2)	38 (2)	153 (1.1)	
Asian	174 (1.1)	18 (<1)	156 (1.1)	
Other	347 (2.2)	51 (2)	296 (2.2)	
Ethnicity, n (**%**)				.23
Non-Hispanic	15,446 (98.1)	2126 (97.7)	13,320 (98.1)	
Hispanic	228 (1.4)	40 (2)	188 (1.4)	
Unknown	76 (<1)	9 (<1)	67 (<1)	
Marital status, n (**%**)				<.001
Married	10,095 (64.1)	1267 (58.3)	8828 (65.0)	
Nonmarried	5655 (35.9)	908 (41.7)	4747 (35.0)	
Single	1724 (10.9)	268 (12.3)	1456 (10.7)	
Divorced	1534 (9.7)	235 (10.8)	1299 (9.6)	
Widowed	2213 (14.1)	383 (17.6)	1830 (13.5)	
Others or unknown	184 (1.2)	22 (1)	162 (1.2)	
Education, n (**%**)				.62
Less than high school	325 (2.1)	53 (2)	272 (2.0)	
High school or some college/associates	5372 (34.1)	742 (34.1)	4630 (34.1)	
College and higher	8115 (51.5)	1112 (51.1)	7003 (51.6)	
Unknown	1938 (12.3)	268 (12.3)	1670 (12.3)	
Language, n (**%**)				
English	15,426 (97.9)	2127 (97.8)	13,299 (98)	.61
Non-English	263 (1.7)	41 (2)	222 (1.6)	
Unknown	61 (<1)	7 (<1)	54 (<1)	
Insurance type, n (**%**)				<.001
Medicare	12,202 (77.5)	1609 (74.0)	10,593 (78.0)	
Commercial	3031 (19.2)	484 (22.3)	2547 (18.8)	
Other	517 (3.3)	82 (4)	435 (3.2)	
ADI^b^, n (**%**)				<.001
Low (≤20)	8278 (52.6)	1207 (55.5)	7071 (52.1)	
Medium (21-40)	4972 (31.6)	649 (29.8)	4323 (31.8)	
High (≥40)	1821 (11.6)	198 (9.1)	1623 (12.0)	
Unknown	679 (4.3)	121 (5.6)	558 (4.1)	
Cognitive tests, n (**%**)				<.001
Mini-Cog	14,278 (90.7)	1402 (64.5)	12,876 (94.9)	
MoCA^c^	904 (5.7)	566 (26.0)	338 (2.5)	
MMSE^d^	538 (3.4)	183 (8.4)	355 (2.6)	
SLUMS^e^	11 (<1)	9 (<1)	2 (<1)	
AD8^f^	19 (<1)	15 (<1)	4 (<1)	
Number of visits in prior year per patient, median (IQR)	8 (4-16)	15 (8‐27)	8 (4-14)	<.001
Total number of notes	605,177	173,475^g^	431,702	—^h^
Number of notes in prior year per patient, median (IQR)	25 (11‐48)	54 (28‐102)	22 (10‐41)	<.001

a *P* values were calculated using 2-tailed* t *tests for continuous variables and chi-square tests or Fisher exact tests for categorical variables, as appropriate. For race and marital status, P values were calculated based on the major categories (White vs Non-White and married vs nonmarried, respectively); subcategories are presented for descriptive purposes only.

b ADI: Area Deprivation Index.

c MoCA: Montreal Cognitive Assessment.

d MMSE: Mini-Mental State Examination.

e SLUMS: Saint Louis University Mental Status Examination.

f AD8: Ascertain Dementia 8-Item Informant Questionnaire

g Of these notes, 7394 had mention of cognitive concerns.

h Not applicable.

Individuals with cognitive concerns were older (mean 75.0, SD 6.9 y vs 73.2, SD 6.3 y), less likely to be married (1267/2175, 58.3% vs 8828/13,575, 65%), more likely to have commercial insurance (484/2175, 22.3% vs 2547/13,575, 18.8%), and more likely to live in less deprived neighborhoods (low ADI: 1207/2175, 55.5% vs 7071/13,575, 52.1%; high ADI: 198/2175, 9.1% vs 1623/13,575, 12.0%). Sex, race, ethnicity, and education distribution were similar between groups with and without documented cognitive concerns. Consistent with these patterns, unadjusted analyses showed higher odds of concern documentation with older age (crude odds ratio [OR] 1.51 per 10-y increase, 95% CI 1.41‐1.61) and commercial insurance (vs Medicare; OR 1.25, 95% CI 1.12‐1.40), and lower odds with being married (OR 0.75, 95% CI 0.68‐0.82) and with greater neighborhood deprivation (medium vs low ADI: OR 0.88, 95% CI 0.79‐0.97; high vs low ADI: OR 0.71, 95% CI 0.61‐0.84; Table S8 in Multimedia Appendix 1).

Cognitive assessment type differed significantly by cognitive concern status (*P*<.001). Individuals without cognitive concerns were more likely to undergo the Mini-Cog (12,876/13,575, 94.9% vs 1402/2175, 64.5%), whereas those with cognitive concerns were more frequently assessed with the MoCA (566/2175, 26% vs 338/13,575, 2.5%) or MMSE (183/2175, 8.4% vs 355/13,575, 2.6%). Mini-Cog scores were marginally lower in the cognitive concern group (4.7 vs 4.8, *P*<.001). Individuals with cognitive concerns also had a substantially higher number of clinical notes per individual (median 54, IQR 28‐102 vs median 22, IQR 10‐41; *P*<.001).

Compared with individuals without cognitive concerns, those with cognitive concerns had a markedly higher frequency of several neurological conditions (stroke or transient ischemic attack [TIA], Parkinson disease, traumatic brain injury [TBI], and epilepsy) and cardiovascular conditions (hypertension, coronary artery disease, atrial fibrillation, and heart failure), whereas obesity and hyperlipidemia were slightly less common (Table 2). In crude analysis, the strongest associations with cognitive concern documentation were observed for Parkinson disease (OR 6.13, 95% CI 4.37‐8.60), TBI (OR 7.75, 95% CI 5.55‐10.85), stroke or TIA (OR 5.00, 95% CI 4.23‐5.90), and epilepsy (OR 3.70, 95% CI 2.78‐4.90), with additional positive associations for depression (OR 2.53, 95% CI 2.29‐2.79), excessive alcohol use (OR 2.36, 95% CI 1.76‐3.12), and anxiety (OR 1.82, 95% CI 1.65‐2.01; Table S5 in Multimedia Appendix 1). Conversely, obesity (OR 0.83, 95% CI 0.75‐0.92) and hyperlipidemia (OR 0.76, 95% CI 0.68‐0.84) were associated with lower odds, indicating cognitive concerns were less likely to be documented in patients with these comorbidities.

**Table 2.** Clinical characteristics of individuals with normal cognitive test scores, stratified by the presence of cognitive concerns.

Characteristics	Overall (N=15,750)	With cognitive concerns (n=2175)	Without cognitive concerns (n=13,575)	*P* value
Neurological conditions, n (**%**)				
Stroke or TIA^b^	629 (4.0)	264 (12.1)	365 (2.7)	<.001
Parkinson disease	137 (0.9)	67 (3)	70 (<1)	<.001
TBI^c^	141 (0.9)	77 (4)	64 (<1)	<.001
Epilepsy	213 (1.4)	78 (4)	135 (1.0)	<.001
Cardiovascular conditions, n (**%**)				
Hypertension	13,001 (82.5)	1936 (89.0)	11,065 (81.5)	<.001
Coronary artery disease	2543 (16.1)	504 (23.2)	2039 (15.0)	<.001
Atrial fibrillation	1907 (12.1)	399 (18.3)	1508 (11.1)	<.001
Heart failure	835 (5.3)	203 (9.3)	632 (4.7)	<.001
Metabolic and endocrine disorders, n (**%**)				
Type 2 diabetes mellitus	2652 (16.8)	481 (22.1)	2171 (16.0)	<.001
Hypothyroidism	2996 (19.0)	475 (21.8)	2521 (18.6)	<.001
Vitamin B12 deficiency	832 (5.3)	168 (7.7)	664 (4.9)	<.001
Obesity	5084 (32.3)	628 (28.9)	4456 (32.8)	<.001
Hyperlipidemia	12,858 (81.6)	1692 (77.8)	11,166 (82.3)	<.001
Psychiatric conditions, n (**%**)				
Depression	3150 (20.0)	763 (35.1)	2387 (17.6)	<.001
Anxiety disorders	3624 (23.0)	721 (33.1)	2903 (21.4)	<.001
Excessive alcohol use	244 (1.5)	66 (3)	178 (1.3)	<.001
Autoimmune and inflammatory disorders, n (**%**)				
Rheumatoid arthritis	364 (2.3)	67 (3)	297 (2.2)	.01
Infectious diseases, n (**%**)				
Urinary tract infection	1197 (7.6)	262 (12.0)	935 (6.9)	<.001
Upper respiratory infection	823 (5.2)	127 (5.8)	696 (5.1)	.18
Gingivitis	24 (<1)	4 (<1)	20 (<1)	.57
Herpes simplex virus	679 (4.3)	114 (5.2)	565 (4.2)	.03
Epstein-Barr virus	8 (<1)	3 (<1)	5 (<1)	.09
Cytomegalovirus	7 (<1)	3 (<1)	4 (<1)	.06
Streptococcal infection	13 (<1)	4 (<1)	9 (<1)	.09
Staphylococcal infection	26 (<1)	6 (<1)	20 (<1)	.28
COVID-19	3129 (19.9)	510 (23.4)	1619 (19.3)	<.001
Sensory impairment, n (**%**)				
Hearing impairment	2911 (18.5)	536 (24.6)	2375 (17.5)	<.001
Visual impairment	4428 (28.1)	662 (30.4)	3766 (27.7)	.01
Lifestyle, n (%)				
Smoking	990 (6.3)	141 (6.5)	849 (6.3)	.72
CCI^d^, mean (SD)	1.7 (2.4)	2.4 (2.8)	1.6 (2.3)	<.001

b TIA: transient ischemic attack.

c TBI: traumatic brain injury.

d CCI: Charlson Comorbidity Index.

Individuals with subjective cognitive concerns had greater exposure to medications with potential cognitive effects than those without concerns (Table 3). Across most major drug classes, including anticholinergics, benzodiazepines and other sedative-hypnotics, antidepressants, antiepileptics, opioids, and other medications of concern such as H2-receptor antagonists, beta-blockers, corticosteroids, and chemotherapy agents, individuals with cognitive concerns were significantly more likely to have at least one prescription. Subclasses that were rarely prescribed (eg, barbiturates and antiparkinsonian anticholinergics) showed no significant differences between groups.

**Table 3.** Medication characteristics of individuals with normal cognitive test scores, stratified by the presence of cognitive concerns.

Characteristics	Overall (N=15,750)	With cognitive concerns (n=2175)	Without cognitive concerns (n=13,575)	*P* value
Anticholinergic medications, n (**%**)				
Urinary antispasmodics	501 (3.2)	124 (5.7)	377 (2.8)	<.001
Antihistamines (first generation)	1121 (7.1)	284 (13.1)	837 (6.2)	<.001
Antimuscarinics (GI^a^)	1750 (11.1)	323 (14.9)	1427 (10.5)	<.001
Skeletal muscle relaxants	1159 (7.4)	256 (11.8)	903 (6.7)	<.001
Benzodiazepines and sedative-hypnotics, n (**%**)				
Benzodiazepines	2520 (16.0)	553 (25.4)	1967 (14.5)	<.001
Nonbenzodiazepine sedative-hypnotics	802 (5.1)	180 (8.3)	622 (4.6)	<.001
Barbiturates	15 (<1)	9 (<1)	6 (<1)	.44
Antidepressants, n (**%**)				
TCAs^b^	340 (2.2)	93 (4.3)	247 (1.8)	<.001
SSRIs^c^	2356 (15.0)	525 (24.1)	1831 (13.5)	<.001
SNRIs^d^	635 (4.0)	207 (9.5)	428 (3.2)	<.001
Mirtazapine	287 (1.8)	101 (4.6)	186 (1.4)	<.001
Antipsychotics, n (**%**)				
Typical antipsychotics (first generation)	908 (5.8)	213 (9.8)	695 (5.1)	<.001
Atypical antipsychotics (second generation)	223 (1.4)	107 (4.9)	116 (0.9)	.55
Antiepileptics, n (**%**)				
Older generation antiepileptics	107 (0.7)	41 (2)	66 (<1)	.02
Newer generation antiepileptics	1657 (10.5)	452 (20.8)	1205 (8.9)	<.001
Opioids, n (**%**)	4063 (25.8)	857 (39.4)	3206 (23.6)	<.001
Other medications of concern, n (**%**)				
Anticholinergic antiparkinsonians	11 (<1)	4 (<1)	7 (<1)	.37
H2^e^-receptor antagonists	1478 (9.4)	338 (15.5)	1140 (8.4)	<.001
Beta-blockers	4066 (25.8)	737 (33.9)	3329 (24.5)	<.001
Corticosteroids	3594 (22.8)	687 (31.6)	2907 (21.4)	<.001
Chemotherapy agents	592 (3.8)	138 (6.3)	454 (3.3)	<.001

a GI: gastrointestinal.

b TCA: tricyclic antidepressant.

c SSRI: selective serotonin reuptake inhibitor.

d SNRI: serotonin-norepinephrine reuptake inhibitor.

e H2: histamine-2.

In multivariable models that are sequentially adjusted for sociodemographic and documentation opportunity factors (model 1), then additionally for clinical comorbidities (model 2), and finally for relevant medication prescriptions (model 3), most associations were attenuated but remained significant (Multimedia Appendix 1 Table S5). Figure 2 shows the aORs and 95% CIs from the fully adjusted model (model 3) across sociodemographics, documentation opportunity, clinical comorbidities, and medication classes. In the fully adjusted model (model 3), among the sociodemographics factors, older age (aOR 1.27 per 10-y increase, 95% CI 1.17‐1.37) and commercial insurance (vs Medicare; aOR 1.35, 95% CI 1.19‐1.53) remained strongly associated with cognitive concern documentation, whereas higher neighborhood deprivation was attenuated (medium vs low ADI: aOR 0.87, 95% CI 0.78‐0.98; high vs low ADI: aOR 0.79, 95% CI 0.66‐0.95). As expected, the number of notes was strongly associated with the documentation of cognitive concerns across all models. Neurological conditions showed the strongest associations, including Parkinson disease (aOR 5.29, 95% CI 3.60‐7.77), TBI (aOR 4.63, 95% CI 3.15‐6.81), stroke or TIA (aOR 3.46, 95% CI 2.86‐4.18), and epilepsy (aOR 2.52, 95% CI 1.83‐3.49). Depression (aOR 1.54, 95% CI 1.36‐1.75) and excessive alcohol use (aOR 1.48, 95% CI 1.06‐2.06) also remained significant. Obesity (aOR 0.73, 95% CI 0.65‐0.82) and hyperlipidemia (aOR 0.59, 95% CI 0.51‐0.67) were associated with lower odds of documentation.

**Figure 2.**
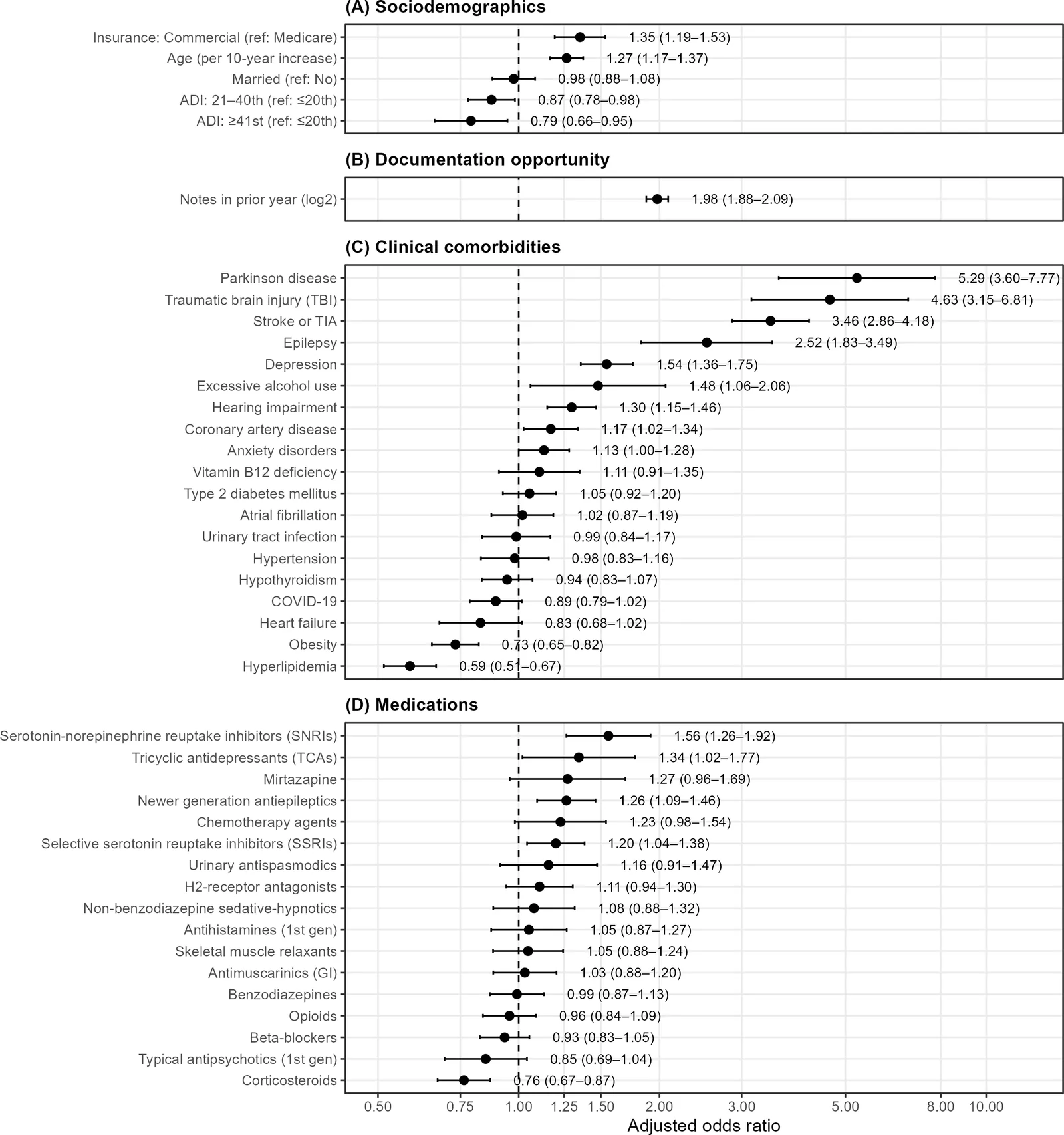
Factors associated with documentation of cognitive concerns in cognitively unimpaired older adults (N=15,750): (A) sociodemographics, (B) documentation opportunity, (C) clinical comorbidities, and (D) medications. GI: gastrointestinal; TIA: transient ischemic attack.

## Discussion

### Principal Findings

In this study, we examined how cognitive concerns are documented in routine clinical care among older adults with normal cognitive test scores. Using EHR data from a large cohort of individuals aged 65 years or older with an initial normal cognitive assessment, we applied an LLM-based NLP pipeline to identify cognitive concerns from clinical notes in the year prior to testing and used multivariable logistic regression to evaluate the association of cognitive concerns with sociodemographic and clinical factors. We found that cognitive concerns were documented in a minority of individuals and within a very small fraction of notes. Several neurologic (Parkinson disease, TBI, stroke or TIA, and epilepsy) and psychiatric (depression, anxiety, and excessive alcohol use) conditions were associated with higher odds of documentation of cognitive concerns, whereas some metabolic conditions (hyperlipidemia and obesity) and living in more deprived neighborhoods (higher ADI) were associated with lower odds. These associations largely persisted after adjustment for sociodemographic factors, documentation opportunity, comorbidities, and relevant medications. By linking structured cognitive test scores with concerns extracted from unstructured notes at scale, this work provides novel insights into how early cognitive symptoms are selectively recognized and recorded in the EHR, with important implications for both clinical care and EHR-based phenotyping.

The frequency of documented cognitive concerns prior to a normal test was low (13.8% overall). Documentation of cognitive concerns was even less common for individuals assessed with the Mini-Cog, which is a very brief cognitive screening test, than for those assessed with other instruments (MoCA, MMSE, SLUMS, and AD8), most of which are longer cognitive screening tests. This suggests that many cognitive tests are performed without an explicitly documented concern, which may reflect routine screening, clinician-initiated evaluation, or underdocumentation of symptoms. Additionally, it suggests that for individuals with cognitive concerns, a longer cognitive screening test is usually used.

Among sociodemographic factors, we observed clear patterns in documentation of cognitive concerns. Older age was associated with higher odds of documentation, consistent with greater symptom burden and increased clinician vigilance in older adults. Individuals with commercial insurance remained more likely to have cognitive concerns documented than those with Medicare, even in the fully adjusted model. This pattern is consistent with prior work showing that privately insured individuals are more likely to receive preventive services and certain types of outpatient care than publicly insured individuals, which may increase opportunities for patients to report and clinicians to document cognitive concerns [18]. In addition, our finding that higher neighborhood deprivation was associated with lower odds of documented cognitive concerns contrasts with prior work linking more disadvantaged neighborhoods to a higher risk of cognitive decline and dementia [19,20]. However, this discrepancy may reflect differential recognition or documentation of early cognitive symptoms in routine care rather than a truly lower burden of cognitive problems. Individuals from more disadvantaged communities may be less likely to access evaluation at earlier stages and may present for care at later stages of cognitive impairment, when deficits are more apparent [10,11]. Thus, lower documentation of subjective concerns among residents of high-ADI neighborhoods may indicate delayed help-seeking, competing clinical priorities, and fewer opportunities for symptom elicitation and recording [10].

Clinical comorbidities showed a coherent pattern. Neurological conditions, such as Parkinson disease, TBI, stroke or TIA, and epilepsy, had the highest adjusted odds, indicating that clinicians are particularly likely to document cognitive concerns when clear neurologic risk factors are present. Depression and excessive alcohol use were also associated with higher odds, likely reflecting overlapping symptoms (eg, memory complaints and concentration) with cognitive concerns and more frequent discussion of cognition in mental health-focused encounters. Hearing impairment showed a modest but consistent association, which may reflect either shared vulnerability or clinical “diagnostic uncertainty,” as hearing-related communication difficulties can prompt concerns about cognition. In contrast, obesity and hyperlipidemia were linked to lower odds of documentation, which may reflect competing clinical priorities, underrecognition of cognitive issues in individuals seen primarily for metabolic disease, and residual confounding, and should be interpreted cautiously pending further study. Other cardiovascular and systemic conditions, including coronary artery disease, showed small positive associations, while several conditions (eg, hypertension and type 2 diabetes) were not independently associated after adjustment. The association for anxiety was attenuated after medication adjustment and became borderline, suggesting the crude comorbidity association may be partly explained by medication use.

Individuals with documented cognitive concerns were also more likely to receive medications with potential cognitive effects, including anticholinergics, benzodiazepines and sedative-hypnotics, antidepressants, antiepileptics, and opioids. These associations may reflect several overlapping mechanisms, including medication-related cognitive symptoms, greater psychiatric and neurological comorbidity burden, and increased clinical attention to cognition among medically complex patients. Because this study focused on documentation of cognitive concerns rather than causal cognitive effects, these findings should not be interpreted as evidence that these medications directly caused cognitive decline. Rather, they highlight the complex clinical context in which cognitive concerns are elicited and documented in routine care.

### Strengths

This study is among the first to use LLMs and a large, well-defined cohort of older adults with normal cognitive test scores to examine patterns of subjective cognitive concern documentation before clear cognitive impairment or diagnosed dementia. We applied a 2-tier LLM-based NLP pipeline with high sensitivity and positive predictive value to identify cognitive concerns from unstructured notes, demonstrating the feasibility of scalable, accurate phenotyping beyond structured data alone. We also integrated rich EHR data, including sociodemographic characteristics, neighborhood deprivation (ADI), detailed comorbidities, and medication prescriptions, enabling a nuanced examination of how clinical and contextual factors jointly affect documentation of SCD. By estimating multiple adjusted models and explicitly focusing on documentation of cognitive concerns rather than test scores or diagnosis, our work offers a novel perspective on how early cognitive symptoms are recognized and recorded in routine care and how this may shape EHR-based research and clinical tools.

### Limitations

This study has several limitations. First, we identified the cohort using cognitive test results recorded in flowsheets; individuals whose assessments were documented only in narrative notes may have been missed, introducing selection bias. Second, “normal” cognition was defined using standard screening cutoffs without adjustments. Because these instruments differ in sensitivity and can be biased by educational and sociodemographic factors, subtle impairment may have been misclassified as normal. Third, cognitive concern status may have been misclassified, potentially underestimating differences between groups: we relied on documented concerns, so concerns that were not elicited, reported, or recorded could have been misclassified as “no concern.” In addition, we restricted NLP extraction to notes within 1 year before the first qualifying cognitive test, potentially missing concerns documented earlier or outside this window. Because cognitive concerns could be documented repeatedly across multiple encounters and specialties over time, the current study was not designed to evaluate longitudinal care pathways or delays between initial concern documentation and cognitive testing. We also did not explicitly model the temporal ordering of neurological conditions, medication exposure, cognitive concern documentation, and cognitive testing. Some neurological diagnoses and medication exposures may have preceded concern documentation, whereas others may have occurred afterward as part of ongoing clinical evaluation and management. Therefore, observed associations should be interpreted as correlates of cognitive concern documentation rather than evidence regarding causal or prognostic sequencing. Future longitudinal studies should evaluate whether EHR-documented cognitive concerns provide incremental predictive value beyond established neurological and clinical risk factors, particularly when concern documentation precedes the onset or recognition of these conditions. Although our two-tier LLM-based pipeline showed high sensitivity and PPV in cognitive concern extraction, some misclassification is inevitable. Because GPT-4o was applied only to Med42-v2-8B positive note chunks, true positive cases missed at the first screening stage could not be recovered in the second stage. Exploratory analysis identified 2 additional reference-standard positive cases among Med42-v2-8B negative cases, suggesting that direct application of GPT-4o to all note chunks could further improve sensitivity if computational resources permit. However, the staged design substantially reduced computational cost and token usage while maintaining strong overall performance. Finally, this single-health-system, cross-sectional EHR study may have limited generalizability and cannot support causal inference or assessment of subsequent cognitive decline and dementia.

### Conclusions

This study used EHR data and LLMs to identify and characterize SCD, showing that cognitive concerns are infrequently documented among older adults with normal cognitive test scores. Documentation was more common in those with neurological, psychiatric, and substance-use conditions, but less common among individuals living in more deprived neighborhoods and those with certain metabolic conditions. These findings suggest inconsistent recognition and documentation of early symptoms, with implications for equity and for EHR-based phenotypes and prediction models. Future work will evaluate whether baseline SCD predicts subsequent cognitive decline, to support more consistent, data-driven early detection and management.

## Supplementary material

10.2196/93011 Multimedia Appendix 1 Supplementary tables on large language model–based cognitive concern extraction, model evaluation, clinical and medication-related factors, cognitive assessment characteristics, and factors associated with documentation of cognitive concerns.

## Abbreviations

ADI: Area Deprivation Index
aOR: adjusted odds ratio
EDW: Enterprise Data Warehouse
EHR: electronic health record
HIPAA: Health Insurance Portability and Accountability Act
*ICD*: *International Classification of Diseases*
LLM: large language model
MCI: mild cognitive impairment
MGB: Mass General Brigham
MMSE: Mini-Mental State Examination
MoCA: Montreal Cognitive Assessment
NLP: natural language processing
OR: odds ratio
PPV: positive predictive value
RPDR: Research Patient Data Registry
SCD: subjective cognitive decline
SLUMS: Saint Louis University Mental Status
TBI: traumatic brain injury
TIA: transient ischemic attack
