# Supplementary material for: Large Language Model–Based Identification of Subjective Cognitive Decline in Electronic Health Records Among Older Adults With Normal Cognitive Testing: Retrospective Cohort Study
Source: JMIR Aging. 2026 Oct 7;9:e93011. doi: 10.2196/93011 (PMC13645317; doi:10.2196/93011)
Supplement: Supplementary file 1 [file aging-v9-e93011-s001.docx]

**Supplementary Online Content**

**Table S1**. Models and prompts for extracting cognitive concerns from clinical notes.

**Table S2**. Medical conditions and ICD codes that are potentially related to subjective cognitive concerns.

**Table S3**. Medications potentially related to cognitive concerns.

**Table S4**. Evaluation of large language models in cognitive concerns extraction.

**Table S5**. Clinical settings of indexed normal cognitive assessments among older adults.

**Table S6**. Clinical settings of cognitive concern documentation in clinical notes.

**Table S7**. Cognitive assessment score distributions by overall and cognitive concern status.

**Table S8**. Factors associated with documentation of cognitive concerns among cognitive unimpaired older adults (N=15,750).

**Table S1**. Models and prompts for extracting cognitive concerns from clinical notes.

| **Model** | **Prompt** |
| --- | --- |
| Med42-v2-8B | system_message = """You are an AI assistant that helps doctors extract specific information from clinical notes regarding cognitive concerns. Your task is to analyze each note carefully and respond only based on the provided text without adding or repeating information.  For each chunk, follow these steps:  1. Determine if a cognitive concern is mentioned (Yes/No).  2. Extract and include only the specific text related to the cognitive concern verbatim from the note. Do not infer or generate additional details beyond the note.  Strictly adhere to the following format:  Cognitive Concern: Yes/No  Concern Text: "[Exact text from the note related to the concern]"  Ensure your response only contains the required information in the specified format."""  template = """<s>[INST] <<SYS>>  {system_message}  <</SYS>>  {user_message} [/INST]""" |
| GPT-4o from Microsoft Azure | system_message = (  "You are an AI assistant that helps doctors extract specific information from clinical notes regarding cognitive concerns."  "Your task is to analyze each note carefully and respond only based on the provided text without adding or repeating information."  )  prompt = f"""  Definition of Cognitive Concern  A cognitive concern refers to subjective complaints or observed cognitive difficulties related to memory, attention, orientation, language, executive function, or self-awareness.  Cognitive concerns may or may not indicate true neurocognitive impairment and can be reported by the patient, family members, caregivers, or clinicians in clinical notes.    Cognitive concerns include:  - Self-reported cognitive complaints (e.g., "I feel more forgetful lately.")  - Family-reported observations (e.g., "My husband struggles to follow conversations.")  - Clinician-noted concerns (e.g., "Patient appears distracted and forgetful during the exam.")  However, not all cognitive concerns indicate cognitive decline. Some concerns may be:  - Mild and stable (e.g., occasional forgetfulness)  - Reversible or temporary (e.g., due to stress, medications, or sleep deprivation)  - Early indicators of progressive decline    Exclusions:  Do not extract cognitive concerns if they result from:  - Altered mental status, delirium, or transient confusion (e.g., infections, medications, or hospitalization).  - Temporary cognitive changes (e.g., post-surgical, medication-related, or due to acute illness).  - Improved cognitive function (e.g., "memory has improved").  - Broad or uncertain mentions without a specific concern (e.g., "cognitive status unclear").    Extraction Instructions  For each chunk:  1. Determine if a cognitive concern is mentioned (Yes/No).  2. Classify the concern as active or historical:  - Active: The concern is ongoing or currently affecting the patient.  - Historical: The concern was mentioned in the past.  3. Identify who reported the concern (e.g., patient, family member such as wife, husband, daughter, son, or caregiver). If unclear, write "unspecified."  4. Extract and include all specific text related to cognitive concerns verbatim from the note. If multiple mentions exist, extract all of them. Do not infer or generate additional details beyond the note.  5. Provide a brief reason for the decision    ---    Response Format  ```  Cognitive Concern: Yes/No  Concern Type: Active/Historical  Reporter: [Reporter type]  Concern Text: "[Exact text from the note related to the concern]"  Reason: "[Brief explanation for classification]"  ```  Here is the clinical note:  {chunk_text}  """  messages = [  {"role": "system", "content": system_message},  {"role": "user", "content": prompt}  ] |

**Table S2**. Medical conditions and ICD codes that are potentially related to subjective cognitive concerns.

| **Medical Conditions** | **ICD codes** | **Simplified rules** |
| --- | --- | --- |
| **Neurological conditions** | | |
| **Stroke or Transient Ischemic Attack (TIA)** | I63.x: Cerebral infarction  I60.x: subarachnoid hemorrhage  I61.x: Intracerebral hemorrhagic  I64: Stroke, not specified as hemorrhage or infarction  G45.x Transient cerebral ischemic attacks and related syndromes (≠ G45.4: transient global amnesia) | Patient is flagged as having stroke or TIA in the past 2 years if:  (1) They had ≥1 encounter with a relevant ICD-10 code, and the encounter date is within the last 2 years, restricting:  - Ischemic stroke (I63.x): search among inpatient encounters all discharge diagnoses.  - ICH and SAH (I60.x, I61.x), search the code among only primary discharge diagnosis.  - TIA: use inpatient and outpatient diagnosis codes.  *OR*  (2) They have a problem list entry with a relevant ICD-10 code (same list), AND the problem was added or updated in the last 2 years.  (Ref: PMID 12364739 [Stroke], 24075778 [TIA]) |
| **Parkinson’s Disease** | G20.x: Parkinson's disease | **≥2 G20.* encounter diagnoses on distinct dates within 5 years**, or an **active problem list entry with G20.*** |
| **Traumatic Brain Injury (TBI)** | S06.x (Intracranial injury) | Acute events with encounter diagnosis codes or new problem list entries within the past 2 years |
| **Epilepsy** | G40.x: Epilepsy and recurrent seizures | ≥ 1 encounter diagnosis within the past 5 years or active problem in the problem list  (Ref: PMID 32683225) |
| **Cardiovascular and Cerebrovascular Conditions** | | |
| **Hypertension** | I10: Essential (primary) hypertension  I15.x. Secondary hypertension | ≥1 diagnosis code  ≥2 BP measurements of ≥140/90 mm Hg,  ≥1 antihypertensive medication  in the past 5 years,  or active problem in the problem list  (Ref: PMID 37696605) |
| **Coronary Artery Disease** | I25.x: Chronic ischemic heart disease | ≥1 encounter diagnosis in the past 5 years  Or active problem in the problem list |
| **Heart Failure** | I50.x: Heart failure | ≥2 encounter diagnosis in the past 5 years  Or active problem in the problem list |
| **Atrial Fibrillation** | I48.x: Atrial fibrillation and flutter | 1 inpatient diagnosis code within the prior 2 year,  2 outpatient codes separated by 7 days within the prior 2 year  Or active problem in the problem list.  (Ref: PMID 35348008) |
| **Metabolic and Endocrine Disorders** | | |
| **Type 2 Diabetes Mellitus** | E11.x: Type 2 diabetes mellitus | Presence of ≥2 ICD-10 code for T2DM in the past 5 years  or, active problem in the problem list.  Exclude patients who had T1DM (ICD 10, E10.X) |
| **Hypothyroidism** | *E03.x*: Other Hypothyroidism | Presence of ≥1 relevant ICD-10 code in the past 5 years  or active problem in the problem list. |
| **Vitamin B12 Deficiency** | *D51.9: Vitamin B12 deficiency anemia, unspecified*  *E53.8: Deficiency of other specified B group vitamins* | Presence of ≥1 relevant ICD-10 code in the past 2 years  or active problem in the problem list. |
| **Obesity** | *E66.x (≠ E66.3): Obesity*  *Z68.3x: Body mass index (BMI) 30-39*  *Z68.4x: BMI 40 or greater, adult* | Include patient if:  (1) ≥2 ICD-10 encounter diagnoses of obesity (E66.x, excluding E66.3) in the past 3 years  OR  (2) Active problem list entry of obesity (E66.x, excluding E66.3) added or updated in past 3 years  OR  (3) Most recent BMI in the past year ≥ 30 kg/m²  AND  Exclude if: Most recent BMI in the past year < 30 (if available) |
| **Hyperlipidemia** | *E78.x (≠E78.6)* | 1. ≥1 encounter diagnosis of ICD-10 code E78.x within the past 2–5 years,  2. Active problem list entry with an E78.x code  3. ≥2 Abnormal lab values related to lipid panel within the past 1 year  (Ref: PMID 28469428) |
| **Psychiatric Conditions** | | |
| **Depression** | *F32.x – Major depressive disorder, single episode (covers F32.0 to F32.A)*  *F33.x – Major depressive disorder, recurrent (covers F33.0 to F33.9)*  *F34.1 – Dysthymia (persistent depressive disorder)*  *F34.8x – Other persistent mood [affective] disorders*  *F34.9 – Persistent mood [affective] disorder, unspecified*  *F39 – Unspecified mood [affective] disorder* | Presence of ≥1 relevant ICD-10 code in the past 2 years  or active problem in the problem list. |
| **Anxiety Disorders** | *F41.x: Other anxiety disorders* | Presence of ≥1 relevant ICD-10 code in the past 2 years  or active problem in the problem list. |
| **Excessive alcohol consumption** | *F10.1x, Z72.1* | Presence of ≥1 relevant ICD-10 code in the past 2 years  or active problem in the problem list. |
| **Autoimmune and Inflammatory Disorders** | | |
| **Rheumatoid Arthritis (RA)** | *M05.x: Rheumatoid arthritis with rheumatoid factor*  *M06.x: Other rheumatoid arthritis* | **≥1 ICD-10 diagnosis code** of M05.x or M06.x within the past **5 years**, **OR**  **Active problem list** entry with code M05.x or M06.x |
| **Infectious Diseases** | | |
| **Urinary tract infection** | *N39.0: Urinary tract infection, site not specified* | ≥1 diagnosis code from the provided list within 2 years, OR  an active problem list entry |
| **Upper respiratory infection** | *J06.9: Acute upper respiratory infection, unspecified* | ≥1 diagnosis code from the provided list within 2 years, OR  an active problem list entry |
| **Gingivitis** | *K05.0x: Acute gingivitis*  *K05.1x: Chronic gingivitis* | ≥1 diagnosis code from the provided list within 2 years, OR  an active problem list entry |
| **HSV (Herpes Simplex Virus)** | *B00.x: Herpesviral infection*  *A60.x: Anagenital herpesviral (herpes simplex) infections* | ≥1 diagnosis code from the provided list within 5 years, OR  an active problem list entry  OR abnormal labs in the past 3 years |
| **EBV (Epstein-Barr Virus)** | *B27.0x: Gammaherpesviral mononucleosis*  *B27.9x: Infectious mononucleosis, unspecified* | ≥1 diagnosis code from the provided list within 2 years, OR  an active problem list entry |
| **CMV (Cytomegalovirus)** | *B25.9: Cytomegaloviral disease, unspecified* | ≥1 diagnosis code from the provided list within 2 years, OR  an active problem list entry |
| **Strep (Streptococcal Infection)** | *B95.0-B95.5* | ≥1 diagnosis code from the provided list within 2 years, OR  an active problem list entry |
| **Staph (Staphylococcal Infection)** | *B95.6-B95.8* | ≥1 diagnosis code from the provided list within 2 years, OR  an active problem list entry |
| **COVID-19** | *U07.1* | Diagnosis  Labs  Medications: Paxlovid or Remdesivir |
| **Sensory Impairment** | | |
| **Hearing impairment** | *H90.x: Conductive and sensorineural hearing loss*  *H91.x: Other and unspecified hearing loss* | ≥1 diagnosis code from the provided list within 5 years, OR  an active problem list entry |
| **Visual impairment** | *H54.0-H54.7: visual loss*  *H40.x: Glaucoma*  *H25.x: Cataracts*  *H52.4: Presbyopia* | ≥1 diagnosis code from the provided list within 5 years, OR  an active problem list entry |
| **Lifestyle** | | |
| **Smoking** | *Z72.0: Tobacco use, current*  *F17.2x: Nicotine dependence* | ≥1 diagnosis code from the provided list within 5 years, OR  an active problem list entry |

**Table S3**. Medications potentially related to cognitive concerns

| **Medications** | **Specific medications** | **Rationale for Inclusion** |
| --- | --- | --- |
| **1. Anticholinergic Medications** | | Anticholinergics block acetylcholine, impairing memory and cognition, especially in older adults. |
| 1.1 Urinary Antispasmodics | Oxybutynin, Tolterodine, Darifenacin, Solifenacin, Trospium | Common bladder control meds with varying anticholinergic burden; Darifenacin and Solifenacin add specificity. |
| 1.2 Antihistamines (First-generation) | Diphenhydramine, Chlorpheniramine, Hydroxyzine, Promethazine, Meclizine | Sedating antihistamines cross the blood-brain barrier, causing confusion and memory issues. |
| 1.3 Tricyclic Antidepressants (TCAs) | Amitriptyline, Nortriptyline, Imipramine, Clomipramine, Doxepin | TCAs have strong anticholinergic effects; Doxepin is notable for sedation even at low doses. |
| 1.4 Antimuscarinics (GI) | Dicyclomine, Hyoscyamine, Scopolamine | Used for IBS or motion sickness; Scopolamine is particularly potent cognitively. |
| 1.5 Skeletal Muscle Relaxants | Cyclobenzaprine, Methocarbamol, Carisoprodol | Anticholinergic and sedative properties can contribute to cognitive fog. |
| **2. Benzodiazepines and Sedative-Hypnotics** | | **These enhance GABA activity, leading to sedation, memory impairment, and slowed cognition.** |
| 2.1 Benzodiazepines | Diazepam, Lorazepam, Alprazolam, Clonazepam, Temazepam, Midazolam | Vary by half-life (e.g., Diazepam is long-acting, Lorazepam short-acting); all linked to cognitive decline. |
| 2.2 Non-Benzodiazepine Sedative-Hypnotics | Zolpidem, Eszopiclone, Zaleplon | "Z-drugs" for sleep have similar cognitive risks to benzodiazepines, especially in the elderly. |
| 2.3 Barbiturates | Phenobarbital, Secobarbital | Older sedatives with high cognitive risk; less common but still relevant in some contexts (e.g., epilepsy). |
| **3 Antidepressants** | | **Beyond TCAs, other antidepressants can affect cognition via sedation or receptor effects.** |
| 3.1 TCAs | (See 1.3: Amitriptyline, Nortriptyline, Imipramine, Clomipramine, Doxepin) | Repeated here for completeness; sedation and anticholinergic effects are key. |
| 3.2 SSRIs (Selective Serotonin Reuptake Inhibitors) | Paroxetine, Fluoxetine, Sertraline, Citalopram | Paroxetine has mild anticholinergic effects; others may cause subtle cognitive blunting in some cases. |
| 3.3 SNRIs (Serotonin-Norepinephrine Reuptake Inhibitors) | Duloxetine, Venlafaxine | May cause sedation or confusion in high doses or sensitive individuals. |
| 3.4 Mirtazapine | Mirtazapine | Sedating antidepressant; histamine antagonism can impair cognition. |
| **4. Antipsychotics** |  |  |
| 4.1 Typical (First-Generation) | Haloperidol, Chlorpromazine, Fluphenazine | Chlorpromazine has strong anticholinergic and sedative effects; Haloperidol less so but still impactful. |
| 4.2 Atypical (Second-Generation) | Quetiapine, Olanzapine, Risperidone, Clozapine | Quetiapine and Olanzapine are sedating; Clozapine has high anticholinergic burden. |
| **5 Antiepileptics** | |  |
| Older Generation | Phenytoin, Carbamazepine, Valproate, Phenobarbital | Phenytoin and Phenobarbital are notably sedating; Carbamazepine can cause confusion. |
| Newer Generation | Topiramate, Gabapentin, Pregabalin, Lamotrigine | Topiramate is infamous for "Dopamax" effect (word-finding difficulty); Gabapentin can sedate. |
| **6 Opioids** | Morphine, Oxycodone, Hydrocodone, Fentanyl, Tramadol | Opioids cause sedation and cognitive slowing via mu-receptor agonism. All can impair attention and memory; Tramadol also has mild serotonergic effects. |
| **7 Other medications of concern** | |  |
| Anticholinergic Antiparkinsonians | Benztropine, Trihexyphenidyl | Used for Parkinson’s or EPS; strong anticholinergic effects on cognition. |
| H2-Receptor Antagonists | Cimetidine, Ranitidine | Older GI meds with mild CNS penetration; can cause confusion in the elderly. |
| Beta-Blockers | Propranolol, Metoprolol, Atenolol | Lipophilic beta-blockers (e.g., Propranolol) cross the BBB, potentially causing cognitive slowing. |
| Corticosteroids | Prednisone, Dexamethasone | High doses or chronic use linked to memory impairment and mood changes. |
| Chemotherapy Agents | Methotrexate, Cyclophosphamide | "Chemo brain" involves cognitive decline; relevant if studying broader populations. |

**Table S4**. Evaluation of large language models in cognitive concerns extraction.

| **Approach** | **TP** | **FP** | **FN** | **TN** | **Sensitivity**  **95% CI** | **Specificity**  **95% CI** | **PPV**  **95% CI** | **NPV**  **95% CI** | **F1-Score**  **95% CI** |
| --- | --- | --- | --- | --- | --- | --- | --- | --- | --- |
| Med42-v2-8B | 88 | 48 | 2 | 362 | 0.978  (0.922-0.997) | 0.883  (0.848-0.912) | 0.647  (0.561-0.727) | 0.995  (0.980-0.999) | 0.779  (0.714-0.835) |
| GPT-4o | 88 | 7 | 2 | 403 | 0.978  (0.922-0.997) | 0.983  (0.965-0.993) | 0.926  (0.854-0.970) | 0.995  (0.982-0.999) | 0.951  (0.917-0.979) |
| Med42-v2-8B + GPT-4o | 86 | 6 | 4 | 404 | 0.956  (0.890-0.988) | 0.985  (0.968-0.995) | 0.935  (0.863-0.976) | 0.990  (0.975-0.997) | 0.945  (0.908-0.976) |

Abbreviations: TP, true positives; FP, false positives; FN, false negatives; TN, true negatives; PPV, positive predictive value; NPV, negative predictive value; CI, confidence interval.

**Table S5**. Clinical settings of indexed normal cognitive assessments among older adults.

| **Clinical setting** | **Patients, n (%)** |
| --- | --- |
| Hospital/ Rehabilitation | 460 (2.9) |
| Outpatient | 15,085 (95.8) |
| *Primary care* | 14,427 (91.6) |
| *Neurology/geriatrics* | 440 (2.8) |
| *Other specialties** | 218 (1.4) |
| Unknown | 205 (1.3) |

* Other outpatient specialties included occupational therapy, physical therapy, sports medicine, laboratory, infusion therapy, care management, psychiatry, behavioral health, dermatology, endocrinology, and other specialties not otherwise categorized.

**Table S6**. Clinical settings of cognitive concern documentation in clinical notes.

| **Clinical setting*** | **Notes**  (n=7,395) | **Patients**  (n=2,175) |
| --- | --- | --- |
| Hospital/Rehabilitation | 2845 (38.5) | 574 (26.4) |
| Outpatient |  |  |
| *Primary care* | 1963 (26.5) | 1045 (48.0) |
| *Neurology/geriatrics* | 447 (6.0) | 257 (11.8) |
| *Rehabilitation/therapy* | 387 (5.2) | 127 (5.8) |
| *Behavioral health* | 326 (4.4) | 100 (4.6) |
| *Medical specialties* | 295 (4.0) | 215 (9.9) |
| *Cardiovascular* | 158 (2.1) | 121 (5.6) |
| *Oncology* | 134 (1.8) | 65 (3.0) |
| *Surgical specialties* | 83 (1.1) | 54 (2.5) |
| *Ancillary/support services* | 58 (0.8) | 43 (2.0) |
| *Other outpatient specialties* | 322 (4.4) | 199 (9.1) |
| Unknown | 377 (5.1) | 205 (9.4) |

*Clinical setting categories: primary care included family medicine, primary care, internal medicine, and adult medicine. Neurology/geriatrics included neurology, gerontology, and neuropsychology. Rehabilitation/therapy included speech therapy, occupational therapy, physical therapy, and physical medicine and rehabilitation. Behavioral health included psychiatry and behavioral health. Cardiovascular included cardiology. Oncology included medical oncology, radiation oncology, neuro oncology, and psychosocial oncology. Medical specialties included endocrinology, rheumatology, gastroenterology, pulmonology, nephrology, urology, pain medicine, and dermatology. Surgical specialties included neurosurgery, orthopaedic surgery, and general surgery. Ancillary/support services included care management, home health services, laboratory, and infusion therapy.

**Table S7**. Cognitive assessment score distributions by overall and cognitive concern status.

| **Cognitive test scores** | **Overall** | **With cognitive concerns** | **Without cognitive concerns** | ***P*-value** |
| --- | --- | --- | --- | --- |
| Mini-cog score, mean | 4.7 (0.4) | 4.7 (0.5) | 4.8 (0.4) | <0.001 |
| MoCA score, mean | 27.4 (1.3) | 27.4 (1.3) | 27.4 (1.3) | 0.855 |
| MMSE score, mean | 28.4 (1.4) | 27.7 (1.3) | 28.7 (1.3) | <0.001 |
| SLUMS Score, mean | 27.8 (0.8) | 27.7 (0.7) | 28.5 (0.7) | 0.31 |
| AD8 Score, mean | 0.3 (0.5) | 0.3 (0.5) | 0.2 (0.5) | 0.779 |
|  | | | | |

**Table S8**. Factors associated with documentation of cognitive concerns among cognitive unimpaired older adults (N=15,750)

| **Characteristic** | **Crude OR**  **(95% CI)** | **Model 1^a^** | **Model 2^b^** | **Model 3^c^** |
| --- | --- | --- | --- | --- |
| **Socio-demographics** | | | | |
| Age (per 10 year) | 1.51 (1.41, 1.61) | 1.24 (1.16, 1.34)^‡^ | 1.27 (1.17-1.37)^‡^ | 1.27 (1.17, 1.37)^‡^ |
| Marital status (Ref: not married) | -- | -- | -- | -- |
| Married | 0.75 (0.68, 0.82) | 0.93 (0.84, 1.03) | 0.98 (0.88-1.08) | 0.98 (0.88, 1.08) |
| Insurance (Ref: Medicare) | -- | -- | -- | -- |
| Commercial | 1.25 (1.12, 1.40) | 1.40 (1.24, 1.58)^‡^ | 1.37 (1.21, 1.55)^‡^ | 1.35 (1.19, 1.53)^‡^ |
| Other | 1.24 (0.97, 1.57) | 2.28 (1.75, 2.97)^‡^ | 2.27 (1.73, 2.98)^‡^ | 2.32 (1.77, 3.04)^‡^ |
| ADI (national; ref Low ≤20^th^) | -- | -- | -- | -- |
| Medium | 0.88 (0.79, 0.97) | 0.83 (0.75, 0.93)^†^ | 0.88 (0.78, 0.98)* | 0.87 (0.78, 0.98)* |
| High | 0.71 (0.61, 0.84) | 0.71 (0.60, 0.85)^‡^ | 0.79 (0.66, 0.94)^†^ | 0.79 (0.66, 0.95)* |
| Unknown | 1.27 (1.03, 1.56) | 1.27 (1.01, 1.58)* | 1.24 (0.99, 1.57) | 1.24 (0.98, 1.57) |
| **Documentation opportunity** | | | | |
| Notes in prior year (log2) | 2.08 (2.00, 2.16) | 2.07 (1.98, 2.15)^‡^ | 1.97 (1.88, 2.07)^‡^ | 1.98 (1.88, 2.09) ^‡^ |
| **Clinical comorbidities** | | | | |
| Parkinson’s disease | 6.13 (4.37, 8.60) | -- | 5.63 (3.85, 8.23)^‡^ | 5.29 (3.60, 7.77)^‡^ |
| TBI | 7.75 (5.55, 10.85) | -- | 4.63 (3.15, 6.80)^‡^ | 4.63 (3.15, 6.81)^‡^ |
| Stroke or TIA | 5.0 (4.23, 5.90) | -- | 3.54 (2.94, 4.27)^‡^ | 3.46 (2.86, 4.18)^‡^ |
| Epilepsy | 3.70 (2.78, 4.90) | -- | 2.58 (1.87, 3.56)^‡^ | 2.52 (1.83, 3.49)^‡^ |
| Depression | 2.53 (2.29, 2.79) | -- | 1.76 (1.56, 1.97)^‡^ | 1.54 (1.36, 1.75)^‡^ |
| Excessive alcohol use | 2.36 (1.76, 3.12) | -- | 1.50 (1.08, 2.09)* | 1.48 (1.06, 2.06)* |
| Hearing impairment | 1.54 (1.38, 1.72) | -- | 1.29 (1.14, 1.45)^‡^ | 1.30 (1.15, 1.46)^‡^ |
| Coronary artery disease | 1.71 (1.53, 1.90) | -- | 1.15 (1.01, 1.31)* | 1.17 (1.02, 1.34) * |
| Anxiety | 1.82 (1.65, 2.01) | -- | 1.21 (1.08, 1.36)^‡^ | 1.13 (1.00, 1.28)* |
| Vitamin B12 deficiency | 1.63 (1.36, 1.94) | -- | 1.11 (0.91, 1.35) | 1.11 (0.91, 1.35)* |
| Type 2 DM | 1.80 (1.59, 2.03) | -- | 1.08 (0.95, 1.23) | 1.05 (0.92, 1.20) |
| Atrial fibrillation | 1.80 (1.59, 2.03) | -- | 0.97 (0.84, 1.12) | 1.02 (0.87, 1.19) |
| Urinary tract infection | 1.85 (1.60, 2.14) | -- | 1.01 (0.85, 1.19) | 0.99 (0.84, 1.17) |
| Hypertension | 1.84 (1.60, 2.12) | -- | 0.97 (0.82, 1.14) | 0.98 (0.83, 1.16) |
| Hypothyroidism | 1.23 (1.10, 1.37) | -- | 0.95 (0.84, 1.08) | 0.94 (0.83, 1.07) |
| COVID-19 | 1.20 (1.07, 1.34) | -- | 0.88 (0.77, 1.00)* | 0.89 (0.79, 1.02) |
| Heart failure | 2.11 (1.78, 2.48) | -- | 0.83 (0.68, 1.01) | 0.83 (0.68, 1.02) |
| Obesity | 0.83 (0.75, 0.92) | -- | 0.73 (0.65, 0.82) | 0.73 (0.65, 0.82)^‡^ |
| Hyperlipidemia | 0.76 (0.68, 0.84) | -- | 0.73 (0.65, 0.82) | 0.59 (0.51, 0.67)^‡^ |
| **Medications** | | | | |
| Serotonin-norepinephrine reuptake inhibitors (SNRIs) | 3.23 (2.71, 3.83) | -- | -- | 1.56 (1.26, 1.92)^‡^ |
| Tricyclic antidepressants (TCAs) | 2.41 (1.88, 3.06) | -- | -- | 1.34 (1.02, 1.77)* |
| Mirtazapine | 3.51 (2.73, 4.47) | -- | -- | 1.27 (0.96, 1.69) |
| Newer generation antiepileptics | 2.69 (2.39, 3.03) | -- | -- | 1.26 (1.09, 1.46)^†^ |
| Chemotherapy agents | 1.96 (1.60, 2.38) | -- | -- | 1.23 (0.98, 1.54) |
| Selective serotonin reuptake inhibitors (SSRIs) | 2.04 (1.83, 2.28) | -- | -- | 1.20 (1.04, 1.38)* |
| Urinary antispasmodics | 2.12 (1.71, 2.60) | -- | -- | 1.16 (0.91, 1.47) |
| H2-receptor antagonists | 2.01 (1.76, 2.29) | -- | -- | 1.11 (0.94, 1.30) |
| Non-benzodiazepine sedative-hypnotics | 1.88 (1.58, 2.23) | -- | -- | 1.08 (0.88, 1.32) |
| Antihistamines (1st gen) | 2.29 (1.98, 2.63) | -- | -- | 1.05 (0.87, 1.27) |
| Skeletal muscle relaxants | 1.87 (1.61, 2.16) | -- | -- | 1.05 (0.88, 1.24) |
| Antimuscarinics (GI) | 1.48 (1.30, 1.69) | -- | -- | 1.03 (0.88, 1.20) |
| Benzodiazepines | 2.01 (1.81, 2.24) | -- | -- | 0.99 (0.87, 1.13) |
| Opioids | 2.10 (1.91, 2.31) | -- | -- | 0.96 (0.84, 1.09) |
| Beta-blockers | 1.58 (1.43, 1.74) | -- | -- | 0.93 (0.83, 1.05) |
| Typical antipsychotics (1st gen) | 2.01 (1.71, 2.36) | -- | -- | 0.85 (0.69, 1.04) |
| Corticosteroids | 1.69 (1.53, 1.87) | -- | -- | 0.76 (0.67, 0.87)^‡^ |

Abbreviations: aOR: adjusted odds ratio; Ref: reference group; ADI: area deprivation index; TIA: transient ischemic attack; TBI: Traumatic Brain Injury; CCI, Charlson comorbidity index.

^a^ Adjusted for socio-demographic characteristics (e.g., age, marital status, insurance type, and ADI) and documentation opportunity (i.e., log2(no. of notes+1)).

^b^ Additionally adjusted for clinical comorbidities, including the CCI and selected chronic conditions.

^c^ Further adjusted for relevant medication prescriptions, including anticholinergics, antidepressants, antipsychotics, benzodiazepines, antiepileptics, opioids, and other medications with potential cognitive effects.

* p < 0.05

^†^ p < 0.01

^‡^ p < 0.001
